# Artificial neural networks and player recruitment in professional soccer

**DOI:** 10.1371/journal.pone.0205818

**Published:** 2018-10-31

**Authors:** Donald Barron, Graham Ball, Matthew Robins, Caroline Sunderland

**Affiliations:** 1 School of Science, Technology and Engineering, University of Suffolk, Ipswich, United Kingdom; 2 John van Geest Cancer Research Centre, School of Science and Technology, Nottingham Trent University, Nottingham, United Kingdom; 3 Institute of Sport, University of Chichester, Chichester, United Kingdom; 4 Sport, Health and Performance Enhancement Research Centre, School of Science and Technology, Nottingham Trent University, Nottingham, United Kingdom; University of Innsbruck, AUSTRIA

## Abstract

The aim was to objectively identify key performance indicators in professional soccer that influence outfield players’ league status using an artificial neural network. Mean technical performance data were collected from 966 outfield players’ (mean SD; age: 25 ± *4* yr, 1.81 ±) 90-minute performances in the English Football League. ProZone’s MatchViewer system and online databases were used to collect data on 347 indicators assessing the total number, accuracy and consistency of passes, tackles, possessions regained, clearances and shots. Players were assigned to one of three categories based on where they went on to complete most of their match time in the following season: group 0 (n = 209 players) went on to play in a lower soccer league, group 1 (n = 637 players) remained in the Football League Championship, and group 2 (n = 120 players) consisted of players who moved up to the English Premier League. The models created correctly predicted between 61.5% and 78.8% of the players’ league status. The model with the highest average test performance was for group 0 v 2 (U21 international caps, international caps, median tackles, percentage of first time passes unsuccessful upper quartile, maximum dribbles and possessions gained minimum) which correctly predicted 78.8% of the players’ league status with a test error of 8.3%. To date, there has not been a published example of an objective method of predicting career trajectory in soccer. This is a significant development as it highlights the potential for machine learning to be used in the scouting and recruitment process in a professional soccer environment.

## Introduction

In 2010, UEFA introduced new Club Licensing and Financial Fair Play Regulations to counteract increasing financial losses and mismanagement within European soccer [1]. Elite clubs in England have extended scouting networks world-wide, taken advantage of new technology for video analysis, developed database systems for player reports and added objective analytics to improve their recruitment policies [2]. This modernizing of the scouting and recruitment process has been an attempt to reduce the losses from player trading. The evolution of scouting practises and the early identification of talented players has also been required due to its link with overall success in professional soccer.

Factors associated with success in soccer have been researched over several decades [3]. Early research into playing success was led by sport scientists and focused on identifying the physical demands of professional soccer across Europe [4]. Despite the wealth of research that has been carried out into the physical demands of match performance, it has become increasingly clear that the area does not offer the key to differentiating between successful and unsuccessful teams and players [4, 5]. Considerable research in youth soccer regarding talent identification has also focused on the anthropometric and physiological aspects of performance [6]. Youth academies have been criticised for a maturational focus in talent identification rather than a skills and development focus [6, 7]. This criticism has been due to a systematic bias in soccer academies around the world towards physically mature players born early in selection years, known as the ‘relative age effect’[6, 7].

Following on from the research into the physical activity of players, there has been an increasing interest in developing profiles of performance involving technical factors. Research into technical factors, just as in physical parameters, have found clear positional differences [3]. The research into playing success so far has supported a greater understanding of soccer as a sport but the research to date has only just scratched the surface. Most of the research has assessed a limited number of variables without any explanation for those selected. If there has been a justification given for the variables used, it has either been due to subjective selection [8], or they have looked to replicate variables used in other studies [9]. Large numbers of variables have been dismissed and have not been explored, leaving a considerable number of research areas still untouched. Insights from the differences between players at various levels and in different playing positions are of great importance as they could be useful in assessments of playing talent for scouting purposes. To the authors’ knowledge there has not been an objective study carried out to develop a predictive model that could support the scouting and recruitment process in soccer.

Much of the previous research in soccer has been carried out using traditional statistical techniques such as regression and discriminant analysis [8, 10, 11]. As performance analysis research has progressed, interest has developed in modelling performance using more advanced statistical techniques. In other fields, artificial neural networks are becoming an increasingly popular alternative to traditional statistical techniques [12]. Artificial neural networks are based on the structure and functionality of the human brain and their main areas of use are in classification and prediction [13, 14]. They are becoming increasingly popular due to their ability to solve real world problems, identify trends in complex non-linear data sets and they do not rely on the data being normally distributed [13, 15].

Artificial neural networks have only just started to be explored as a method of analysing performance data in team sports and they offer a novel approach to predicting the career trajectory of professional footballers. There is currently a dearth of research tracking the movement of players between playing levels and the objective performance data that contributes to their career trajectory. By assessing a vast number of variables objectively for a larger sample size than previously used within the existing literature, it is hoped that the key factors linked with career progression can be established. Thus, providing a valuable tool to support the assessment of potential transfer targets in professional soccer and build on the subjective assessments of coaches and scouts. Therefore, the aim of the current study was to develop an objective model to identify key performance indicators in professional soccer that influence outfield players’ league status using an artificial neural network.

## Materials and methods

### Players and match data

Technical performance data and biographical data (mean SD; age and height: 25 ± *4* years, 1.81 ± *0*.*06* m) was collected on 966 outfield players, each completing the full 90 minutes from 1104 matches played in the English Football League Championship during the 2008/09 and 2009/10 seasons. ProZone’s MatchViewer software (ProZone Sports Ltd., Leeds, UK) was used to compile 335 performance variables, including the total number, accuracy (% success), means, medians and upper and lower quartiles of passes, tackles, possessions regained, clearances and shots. The ProZone MatchViewer system used to collect performance data provides five key variables on actions performed during a match; event, time of event, player one involved and player two involved (if relevant) [16]. The system has been shown to have good inter-observer agreement for the number and type of events, the first player involved in events and for the second player involved (k > 0.9) [16].

The data set originally included 505 variables but those with low variance were removed. The data collected for analysis was made available by STATS LLC (Chicago, USA). The official Football League (www.efl.com) and Scout7 Ltd (Birmingham, UK) websites were used to collect additional data on 12 variables including total appearances, playing percentage, total goals and assists, international appearances and heights. Each players’ match by match data for the 335 performance variables was converted into a mean to represent their average 90 minute performance before they were assigned to categories. Institutional ethical approval was attained from the Non-Invasive Human Ethics Committee at Nottingham Trent University.

### Player grouping

Players were assigned to one of three categories based on where they went on to complete most of their match time during the following season. Table 1 provides an outline of the biographical data for the players within the three different categories. The first category included the players who completed most of their match time in a lower league during the following season (Group 0: n = 209 and mean 90 minute appearances = 10 ± *10)*. The second group included those players who completed most of their match time in the English Football League Championship during the following season (Group 1: n = 637 and mean 90 minute appearances = 18 ± *12*). The final category contained the players who progressed to complete most of their match time in the English Premier League during the following season (Group 2: n = 120 and mean 90 minute appearances = 19 ± *12*). Sample sizes for each comparison were balanced to have an equal number of cases using a random number selector (i.e. 209 players were randomly selected from group 1 to have an equal number of cases for comparisons to group 0). The three categories were subsequently analysed using a Stepwise Artificial Neural Network approach to identify the optimal collection of variables for predicting playing status. This was achieved by comparing 2 of the 3 groups at a time using the neural network to identify the key variables responsible for the players’ league status.

**Table 1:** Biographical data represented as means and standard deviations for player groupings.

Variables	Group 0	Group 1	Group 2
N	209	637	120
Age	25.5 ± *4*.*8*	25.4 ± *3*.*9*	25.6 ± *3*.*9*
Height	181.6 ± *5*.*9*	181.0 ± *6*.*1*	181.4 ± *5*.*5*
90 Minute Appearances	10 ± *10*	18 ± *12*	19 ± *12*
Total Minutes	1262.9 ± *1014*.*4*	2048.4 ± *1044*.*6*	2223.7 ± *1132*.*5*

### Artificial neural network model

The artificial neural network modelling was based on the approach previously used successfully in gene profiling with breast cancer data [15]. Prior to artificial neural network training, the data was randomly split into three subsets; 60% for training purposes, 20% for validation and 20% to independently test the model on blind data. The procedure used a Monte-Carlo cross validation procedure that has been shown to outperform and be more consistent than other methods such as the leave-one out cross validation [15]. It also serves the benefit of avoiding over fitting of the data. The artificial neural network modelling involved a multi-layer perceptron architecture with a back-propagation algorithm. This algorithm used a sigmoidal transfer function and weights were updated by feedback from errors.

The learning rate (the rate at which weights are updated as a proportion of the error) was set at 0.1 while the momentum (the proportion of the previous change in weights applied back to the current change in weights) was 0.5. Two hidden nodes (feature detectors) were used as part of the artificial neural network architecture in a single hidden layer. The maximum number of epochs (updates of the network) used was 300 while the maximum number of epochs without improvement on the test was 100. This was used to prevent over fitting of the model. Results were provided for the average test performance and the average test error. The average test performance indicates the percentage of test cases that are correctly predicted. The average test error is the root mean square error for the test data set, which indicates the difference between the values predicted by the model and the actual values of the test data set [17].

## Results

Analysis using the artificial neural network did not provide a suitable model to detect the differences between players in group 0 and group 1. The best model produced by the neural network for group 0 v 1 correctly predicted 67.9% of the test group players’ playing status with an error of 10.8% using a combination of nine variables. The first two variables identified by the model were playing percentage (Group 0 = 30.5 ± *24*.*5*, group 1 = 49.5 ± *25*.*2*) and percentage of backwards passes successful (Minimum) (Group 0 = 66.3 ± *38*.*6*, group 1 = 52.9 ± *38*.*3*). Table 2 provides the results of the model for the group 0 and group 1 comparison and details of the descriptive statistics of the model variables. The neural network did not find a suitable model to detect the differences between those players in group 1 and group 2, results for this comparison can be seen in Table 3. The best model produced by the neural network for group 1 v 2 correctly predicted 61.5% of the test group players’ playing status with an error of 11.6% using a combination of seven variables.

**Table 2:** Results for group 0 v group 1 balanced data set (Best Average Test Performance = 67.9% and Best Average Test Error = 10.8% with a combination of nine variables) and group 0 v group 1 model variables as means and standard deviations for player groupings.

Rank	Variable	Average Test Performance (%)	Average Test Error (%)	Group 0 Means and Standard Deviations	Group 1 Means and Standard Deviations
1	Playing %	65.5	11.2	30.5 ± *24*.*5*	49.5 ± *25*.*2*
2	% of Backwards Passes Successful (Minimum)	65.5	11.0	66.3 ± *38*.*6*	52.9 ± *38*.*3*
3	Total Assists	66.7	10.9	0.9 ± *1*.*6*	1.7 ± *2*.*1*
4	% of Forwards Passes Successful (Median)	66.7	10.9	56.3 ± *14*.*2*	56.9 ± *11*.*5*
5	Total Shots on Target (Excluding Blocked) (Mean)	66.7	10.9	0.3 ± *0*.*4*	0.4 ± *0*.*5*
6	Offsides (Mean)	66.7	10.9	0.3 ± *0*.*7*	0.3 ± *0*.*6*
7	Shots On Target Outside the Box (Maximum)	66.7	10.8	0.8 ± *0*.*8*	1.3 ± *1*.*1*
8	Long Passes (Maximum)	67.9	10.9	9.0 ± *5*.*3*	10.9 ± *6*.*1*
9	First Time Passes Unsuccessful (Upper Quartile)	67.9	10.8	3.1 ± *1*.*5*	3.2 ± *1*.*5*
10	Passes Successful Own Half (Lower Quartile)	66.7	10.8	6.6 ± *5*.*0*	6.2 ± *4*.*3*

**Table 3:** Results for group 1 v group 2 balanced data set (Best Average Test Performance = 61.5% and Best Average Test Error = 11.6% with a combination of seven variables) and group 1 v group 2 model variables as means and standard deviations for player groupings.

Rank	Variable	Average Test Performance (%)	Average Test Error (%)	Group 1 Means and Standard Deviations	Group 2 Means and Standard Deviations
1	% Unsuccessful Headers (Lower Quartile)	54.2	12.3	44.2 ± *14*.*5*	40.7 ± *16*.*6*
2	Number of Possessions (Median)	56.3	12.2	44.3 ± *8*.*8*	46.4 ± *8*.*2*
3	Interceptions (Mean)	56.3	12.2	14.3 ± *7*.*9*	14.0 ± *8*.*5*
4	Total Blocked Shots (Maximum)	55.2	12.2	1.5 ± *1*.*0*	1.5 ± *1*.*1*
5	Total Goals	55.2	12.0	2.6 ± *3*.*4*	4.6 ± *5*.*2*
6	Crosses (Upper Quartile)	59.4	11.6	2.1 ± *1*.*9*	2.2 ± *2*.*0*
7	Total Blocked Shots (Mean)	61.5	11.6	0.4 ± *0*.*4*	0.3 ± *0*.*3*
8	First Time Passes Successful (Upper Quartile)	60.4	11.6	7.6 ± *3*.*5*	8.2 ± *3*.*5*
9	% Successful Headers (Lower Quartile)	59.4	11.6	30.7 ± *14*.*0*	30.9 ± *14*.*5*
10	Average Touches (Maximum)	60.4	11.6	2.4 ± *0*.*9*	2.4 ± *0*.*6*

The most prominent variables in the model were percentage unsuccessful headers (Lower quartile) (Group 1 = 44.2 ± *14*.*5*, group 2 = 40.7 ± *16*.*6*) and number of possessions (Median) (Group 1 = 44.3 ± *8*.*8*, group 2 = 46.4 ± *8*.*2*). Full details can be seen for descriptive statistics of the model variables in Table 3. However, it did find a strong model for distinguishing between players in group 2 and group 0, the results for this comparison can be seen in Table 4. The best model produced by the neural network for group 0 v 2 correctly predicted 78.8% of the test group players’ playing status with an error of 8.3% using a combination of ten variables. U21 caps (Group 0 = 0.9 ± *2*.*7*, group 2 = 3.0 ± *4*.*9*), senior international caps (Group 0 = 3.1 ± *11*.*9*, group 2 = 7.6 ± *14*.*0*) and tackles (Median) (Group 0 = 3.1 ± *1*.*5*, group 2 = 3.0 ± *1*.*2*) were the three most prominent variables in this model. An outline of group means and standard deviations are available in Table 4.

**Table 4:** Results for the group 0 v group 2 balanced data set (Best Average Test Performance = 78.8% Best Average Test Error = 8.3% with a combination of ten variables) and group 0 v group 2 model variables as means and standard deviations for player groupings.

Rank	Input ID	Average Test Performance (%)	Average Test Error (%)	Group 0 Means and Standard Deviations	Group 2 Means and Standard Deviations
1	Under 21 International Caps	69.7	10.2	0.9 ± *2*.*7*	3.0 ± *4*.*9*
2	Full International Caps	71.2	9.5	3.1 ± *11*.*9*	7.6 ± *14*.*0*
3	Tackles (Median)	73.5	9.1	3.1 ± *1*.5	3.0 ± *1*.*2*
4	% First Time Passes Unsuccessful (Upper Quartile)	75.8	8.9	38.3 ± *15*.*5*	36.1 ± *11*.*2*
5	Fouls	75.8	8.8	16.8 ± *16*.*4*	29.1 ± *19*.*7*
6	Dribbles (Maximum)	77.3	8.5	1.2 ± *1*.*2*	2.3 ± *1*.*8*
7	Possession Gained (Minimum)	78.8	8.4	13.4 ± *7*.*5*	10.8 ± *7*.*1*
8	Number of Possessions (Mean)	78.8	8.5	44.0 ± *8*.*5*	46.6 ± *8*.*1*
9	Penalty Area Entries (Median)	78.8	8.6	3.4 ± *2*.*7*	3.7 ± *3*.*0*
10	Average Time in Possession (Maximum)	78.8	8.3	2.9 ± *0*.*4*	3.1 ± *0*.*5*

## Discussion

The aim of the current study was to develop an objective model to identify key performance indicators in professional soccer that influence outfield players’ league status using an artificial neural network. 966 players’ performances were analysed and they were divided into three groups independent of playing position, to highlight key differences between players who went on to play at different levels of the English professional soccer structure. Artificial neural networks were chosen for this research due to their ability to provide highly accurate predictive methods in complex data sets and the issues traditional statistics have dealing with complex non-linear data [14]. They also offer an objective method to identify key performance indicators in contrast to the subjective methods that have typically been used. The artificial neural network model created can accurately detect players that will be promoted to a higher level and those that will play at a lower level. Other comparisons were not accurately predicted by the artificial neural network models.

### Artificial neural network architecture

A constrained architecture with 2 hidden nodes was used and the initial weights were set with a small variance. The purpose of this was to prevent overfitting and eliminate the risk of false discovery and generality. The use of more hidden nodes and hidden layers had the effect of increasing the training time and a loss of performance on the unseen data was observed, indicating loss of generality of the classifiers. The models developed used a Monte Carlo cross validation approach coupled with early stopping and multiple repeats to maximise generality and to also prevent overfitting. Learning rates and momentum were set at 0.1 and 0.5. These only had a minor impact on the performance of the developed classifiers.

### Overview of models

The results from the neural networks did not provide a strong model for group 0 v 1 or group 1 v 2 comparisons. However, a stronger model for comparing players dropping down to a lower playing level compared with those progressing to play in the English Premier League was found with 78.8% of test cases being predicted correctly. These findings would appear logical as the players going on to play in the Premier League and a lower division in the following season should be the furthest apart in playing ability and the neural network performed best at identifying the category of the players in these two groups and the differences between them. The artificial neural network’s ability to correctly classify 78.8% of the player groupings for this model is an important result and it has outperformed other models that have been created to classify performance in cricket [18, 19].

### Key variables in group 0 v group 2 model

#### International experience

The first two factors identified by the model comparing group 2 and group 0 relate to the international experience of the players at Under 21 and senior level. This would indicate that national associations are successful at identifying the most talented players at a young age. It would appear logical that players achieving more international caps would be more successful than their uncapped counterparts. Players moving onto play in the Premier League during the following season averaged the most international caps and U21 caps out of the three groups (Group 0 = 3.13 international caps and 0.93 U21 caps, group 1 = 3.99 international caps and 1.72 U21 caps and group 2 = 7.62 international caps and 3.01 U21 caps). This may also indicate another form of bias being shown by professional clubs towards some players in their selection and recruitment processes. The relative age effect describes the bias towards players born early in selection years, due to their physical maturity, within soccer academies [20]. It could be possible that players within the professional game who achieve international recognition at an early age are looked upon favourably after this point and afforded better opportunities to progress in the future regardless of their current performance levels. These factors can be viewed as esteem or reputation indicators rather than as technical or tactical indicators and they may be currently driving recruitment processes.

#### Defensive variables

The third factor in the model is for the median number of tackles, which also relates to the seventh factor of minimum possessions gained. Players from group 0 had a higher average for median tackles and minimum possessions gained. This is in contrast with the common results of research into these factors. This may be caused by factors specific to the competition the study was conducted from, as previous studies have used samples from international soccer and European competitions. More successful players are thought to read the game and anticipate opposition player’s actions better allowing them to make vital interceptions and tackles [21]. Lago-Penas and Lago-Ballesteros [22], when investigating game location and its effect on results, found that home teams had significantly higher means for gains of possession. More recent research into team success and defensive actions has also shown that the number of tackles had a positive impact on the probability of teams winning matches in the group stages of the 2014 Brazil World Cup [8].

More successful teams have also been shown to have more aggressive approaches to regaining possession through tackles and interceptions, with specific emphasis on regaining the ball in the final third of the pitch [23]. It has become increasingly popular for modern teams to utilise a high pressing approach to their play without possession and prominent coaches such as Pep Guardiola and Jürgen Klopp have had great success using this philosophy [24]. The current study was not able to assess contextual data around the location of regains and tactical approaches which may provide further insights into the defensive variables assessed. Defensive aspects of performance and the role transitions play in match outcomes and player performance have had far less attention from researchers in the analysis of soccer. These are vital areas that warrant far greater focus in the future.

#### Passing variables

The fourth factor from the model regards the percentage of first time passes that are unsuccessful (upper quartile). Players moving onto play in the Premier League during the following season averaged the fewest unsuccessful first time passes out of the three groups (Group 0 = 38.31, group 1 = 39.38 and group 2 = 36.08). Research into the long-term evolution of soccer has shown a considerable increase in passing rates and ball speed over time [25]. Defences have been shown to be more compact in the modern game and effective first time passes are a method of breaking down defences to create scoring opportunities [25]. The current findings may be highlighting that more successful players are better at completing passes and playing at a higher tempo to break down a compact defensive shape.

Previous studies into the success of teams and the differences between players in these teams have highlighted the importance of several passing statistics but first time passes have not been assessed [8, 26]. Their research has not included the depth of technical events and multitude of passing statistics involved in the current study. With the amount of data points now available from computer systems it is important to analyse aspects of play such as passing in greater detail than research has to date. The accuracy for passes over varying distances, in different directions and in key areas of the pitch should be analysed in greater detail. Artificial neural networks are designed specifically for classification and prediction studies where large data sets are involved that may not have obvious linear relationships [13]. This makes them particularly well suited to the sporting context and provides a method for identifying relationships in the data that traditional statistical methods are not suited to analysing.

#### Number of possessions and penalty area entries

Other prominent indicators highlighted by the model included the mean number of possessions and the median penalty area entries. Players moving onto the Premier League averaged the highest mean number of possessions of all the three groups (Group 0 = 43.97, group 1 = 44.83 and group 2 = 46.6). This could indicate that more successful players are involved more in matches, this could be due to them having a better tactical awareness and having better movement off the ball to find space to receive in. Previous studies have identified that players in more successful teams are involved more in matches and receive more passes [5]. They could also be playing in teams that maintain possession better, this is a much-researched area in soccer across several competitions and countries within Western Europe [8, 26]. Some studies have conflicted on the value of possession in relation to team success. However, the most detailed recent investigation into the link between team success and possession has confirmed its strong association with overall success [26]. The paper did also stress that the quality of possession and efficiency factors such as the accuracy of passing and shots were key indicators of a match day performance and not just the total time of possession [26].

A critical aspect of attacking play, which is required for effective possession, is being able to find teammates within the penalty area [27]. Penalty area entries have been shown to differentiate between winning and losing teams. Creating more entries into the opposition penalty area also leads to a higher chance of scoring and allowing fewer penalty area entries means a team is less likely to concede a goal [27]. The model could be indicating that more successful players are better at reading game situations where it is possible to pass the ball into teammates in the penalty area. More skilful players have been shown to be better than their less skilled counterparts at reading patterns of play in matches and monitoring movement off the ball, aiding their decision-making skills [28, 29].

### Study limitations

Although this study represents the first attempt to objectively identify the key indicators driving recruitment in Association Football, there are a couple of limitations to this study that should be addressed in future research. The main limitation was analysing the three discrete groups regardless of playing position. Previous research in England and across European leagues has shown that standard playing profiles vary greatly between different positions in terms of their physical output, their defensive contribution and their involvement in the attacking aspects of a performance [4, 30–32]. It would be logical to assume that positional differences will exist within the Football League Championship due to the research currently available in other leagues and this should be examined further in future research.

The second key limitation involves the lack of information regarding the physical capabilities and performance of the players involved. A wide variety of in-depth physical performance data is currently collected on players’ performances during testing protocols, training sessions and matches. This information was not available to be included in the current study due to the sensitive nature of the data. Previous research has identified that technical indicators have a stronger association with match outcome and team success than physical indicators [33]. However, a players’ ability to meet the physical requirements of matches influences their ability to maintain their technical performance [4]. If this information could be made available and incorporated into the study design, it would improve the scope of the research and may increase the accuracy of the predictive models.

## Conclusions

The findings of this study have shown that it is possible to identify performance indicators using an artificial neural network that influence a players’ league status and accurately predict their career trajectory. A process has also been laid out for further analysis in this area. Future research must build on the current findings through more position specific analysis and by assessing players based on their physical and technical performance to improve the accuracy of such models.

Through further research a process could be developed to accurately predict a players’ future playing status using performance data. This process has previously been largely a subjective process leading to inaccuracies and bias towards variables that do not predict career trajectory. The artificial neural network model could be a crucial objective tool to aid the selection of key players for scouting purposes and to compare and assess transfer targets as part of the recruitment process. Thus, leading to a more efficient and accurate scouting and recruitment process in the future.
